# Bufadienolides originated from toad source and their anti-inflammatory activity

**DOI:** 10.3389/fphar.2022.1044027

**Published:** 2022-10-19

**Authors:** Denglang Zou, Qiqi Wang, Tao Chen, Duocheng Sang, Tingqin Yang, Yuhan Wang, Mengze Gao, Fangfang He, Yulin Li, Liangliang He, Duojie Longzhu

**Affiliations:** ^1^ School of Life Science, Qinghai Normal University, Xining, China; ^2^ College of Pharmacy, Jinan University, Guangzhou, China; ^3^ Northwest Institute of Plateau Biology, Chinese Academy of Sciences, Xining, China

**Keywords:** toad sourced bufadienolide, structure diversity, MS fragmentation principles, anti-inflammatory activity, structure modification

## Abstract

Bufadienolide, an essential member of the C-24 steroid family, is characterized by an α-pyrone positioned at C-17. As the predominantly active constituent in traditional Chinese medicine of Chansu, bufadienolide has been prescribed in the treatment of numerous ailments. It is a specifically potent inhibitor of Na^+^/K^+^ ATPase with excellent anti-inflammatory activity. However, the severe side effects triggered by unbiased inhibition of the whole-body cells distributed α1-subtype of Na^+^/K^+^ ATPase, restrict its future applicability. Thus, researchers have paved the road for the structural alteration of desirable bufadienolide derivatives with minimal adverse effects *via* biotransformation. In this review, we give priority to the present evidence for structural diversity, MS fragmentation principles, anti-inflammatory efficacy, and structure modification of bufadienolides derived from toads to offer a scientific foundation for future in-depth investigations and views.

## Introduction

Bufadienolide, an essential member of the C-24 steroid family, is characterized by an α-pyrone (six-membered lactone) ring positioned at C-17 (Gao et al., 2011). In 1933, the first bufadienolide, namely Scillaren A, was isolated and identified from Egyptian squill (Stoll et al., 1933). Since then, this class of steroid has become a research hotspot, piquing the interest of researchers worldwide, to promote the isolation and identification of bufadienolide with structural diversity (Steyn and van Heerden 1998). Currently, an increasing number of bufadienolides were originally characterized from the traditional Chinese medicine of ChanSu which is one of the richest sources of bufadienolides (Rodríguez et al., 2017; Wei et al., 2019; Pearson and Tarvin 2022). Another notable source is venom from toads (Bufonidae), such as *Bufo bufo gargarizans* Cantor (Zhan et al., 2020), *Rhinella marina* (Hayes et al., 2009), and *Bufo melanostictus* Schneider (Verpoorte and Svendsen 1979; Verpoorte and Svendsen 1980).

Remarkable progress, in terms of pharmacology and the potential of bufadienolide as a therapeutic agent, has been achieved in recent years. Importantly, bufadienolide exhibited significant antitumor and anti-inflammatory activity (Ogawa et al., 2009; Kanai et al., 2013). The pharmacological mechanism behind bufadienolide was the alteration of intracellular calcium concentration, which was triggered by its potent inhibition of Na^+^/K^+^ ATPase (Holmgren et al., 2000). When calcium ion accumulates within the appropriate concentration range in the body, bufadienolide displays the desired biological properties, such as anticancer and anti-inflammatory activity. However, if the calcium ion accumulates in a manner far beyond the threshold concentration, it might result in rather severe toxicity. Brilliantly, some creatures utilized the toxic bufadienolide as chemical defense weapons to highlight its ecological value, which was given by its powerful Na^+^/K^+^ ATPase inhibitory activity. Unarmed animals, such as fireflies and Asian snakes, have been found to sequester and store numerous bufadienolides for defensive purposes (Dobler et al., 2012; Petschenka and Agrawal 2015; Ujvari et al., 2015; Mohammadi et al., 2016; Smedley et al., 2017; Bókony et al., 2018).

Promisingly, an increasing number of researchers have concentrated on the anti-inflammatory properties of bufadienolide in recent years. In the 1960s, the anti-inflammatory activity of bufadienolide was firstly documented (Lancaster and Vegad 1967). After that, numerous investigations have verified the anti-inflammatory effect of bufadienolide, as demonstrated by a reduction in inflammatory symptoms in various animal models of acute and chronic inflammation (Esposito 1985; de Vasconcelos et al., 2011; Fürst et al., 2017). To discover the underlying mechanisms, the majority of studies have focused on leukocytes. It was found that bufadienolide exhibited anti-inflammation activity by primarily inhibiting cell proliferation and the secretion of proinflammatory cytokines (Forshammar et al., 2011; Forshammar et al., 2013).

However, some nonnegligible drawbacks, including high toxicity, low bioavailability, and poor water solubility of bufadienolide, severely restrict its further applications (Deng et al., 2017; Fu et al., 2019; Shao et al., 2021). Hence, it is particularly essential to find effective ways for structural modification of bufadienolide with the potential to both lower toxicity and increase or at least keep activity. Although chemical synthesis is usually the go-to option (Michalak et al., 2017; Shimizu et al., 2020; Wu et al., 2020), it is severely hindered by factors beyond the control, such as chemical waste, tedious protection and deprotection steps, unsatisfied synthesis yield accompanied with undesired by-products. Biosynthesis, on the other hand, has been emerged and recognized as an outstanding alternative to conquer these obstacles due to its eco-friendly and cost-effective qualities (Heasley 2012; Huang et al., 2019; Hoshino and Gaucher 2021; Pavesi et al., 2021). In the field of biosynthesis, glycosylation may be a mainstream method for bufadienolide structure modification (Thorson and Vogt 2003; Zhou et al., 2012). Bufadienolide glycosylation generally refers to the enzyme catalysis process to attach glycans at their C-3 position. It was not until 2007 that Thorson performed the first successful enzyme catalytic glycosylation of cardiac steroids (Williams et al., 2007; Gantt et al., 2008). An actinomycete-sourced glycosyltransferase OleD and its practical mutant ASP with substrate heterogeneity were discovered through high-throughput screening to perform cardiac steroid glycosylation. Accordingly, Wen et al. further discovered that C-3 glycosylation of bufadienolide could be accomplished by a glycosyltransferase called UGT74AN1 in an environmentally friendly and highly effective manner (Wen et al., 2018).

The characters, in terms of attractive structure diversity and promising biological activity, make bufadienolide a hot research topic. In the present review, we primarily analyzed and reviewed the current evidence for structure diversity, MS fragmentation principles, anti-inflammatory activity and structure modification of bufadienolides originated from toad source to provide a scientific basis for future in-depth studies and perspectives of potential drug candidates discovery.

## Structure diversity

**FIGURE 1:**
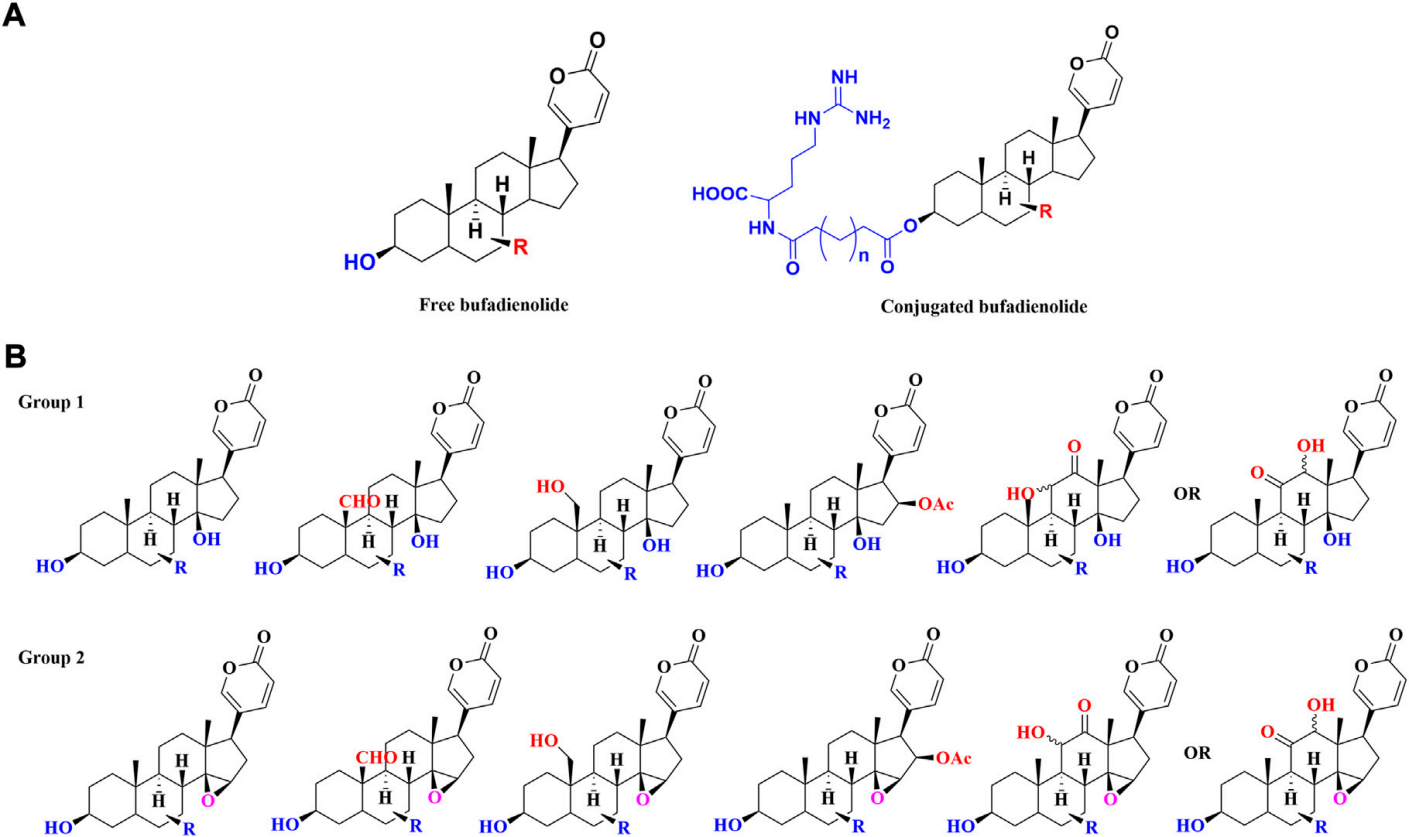
The classification of toad-sourced bufadienolide: **(A)** The typical structures of free bufadienolide and conjugated bufadiendolide; **(B)** The typical structures of five sub-categories of free bufadienolide according to the contained substituents.

Structurally, toad-sourced bufadienolide could be classified into two groups: free bufadienolide and conjugated bufadienolide. The steroid core of free bufadienolide is replaced with a variable amount of hydroxyl groups. For conjugated bufadienolide, it is defined by esterification of hydroxyl group at C-3 position with specific groups, including argininyl side chain, lactic acid and sulfonic acid. The typical structures of free bufadienolide and conjugated bufadienolide were shown in Figure 1A.

### Free bufadienolide

**FIGURE 2:**
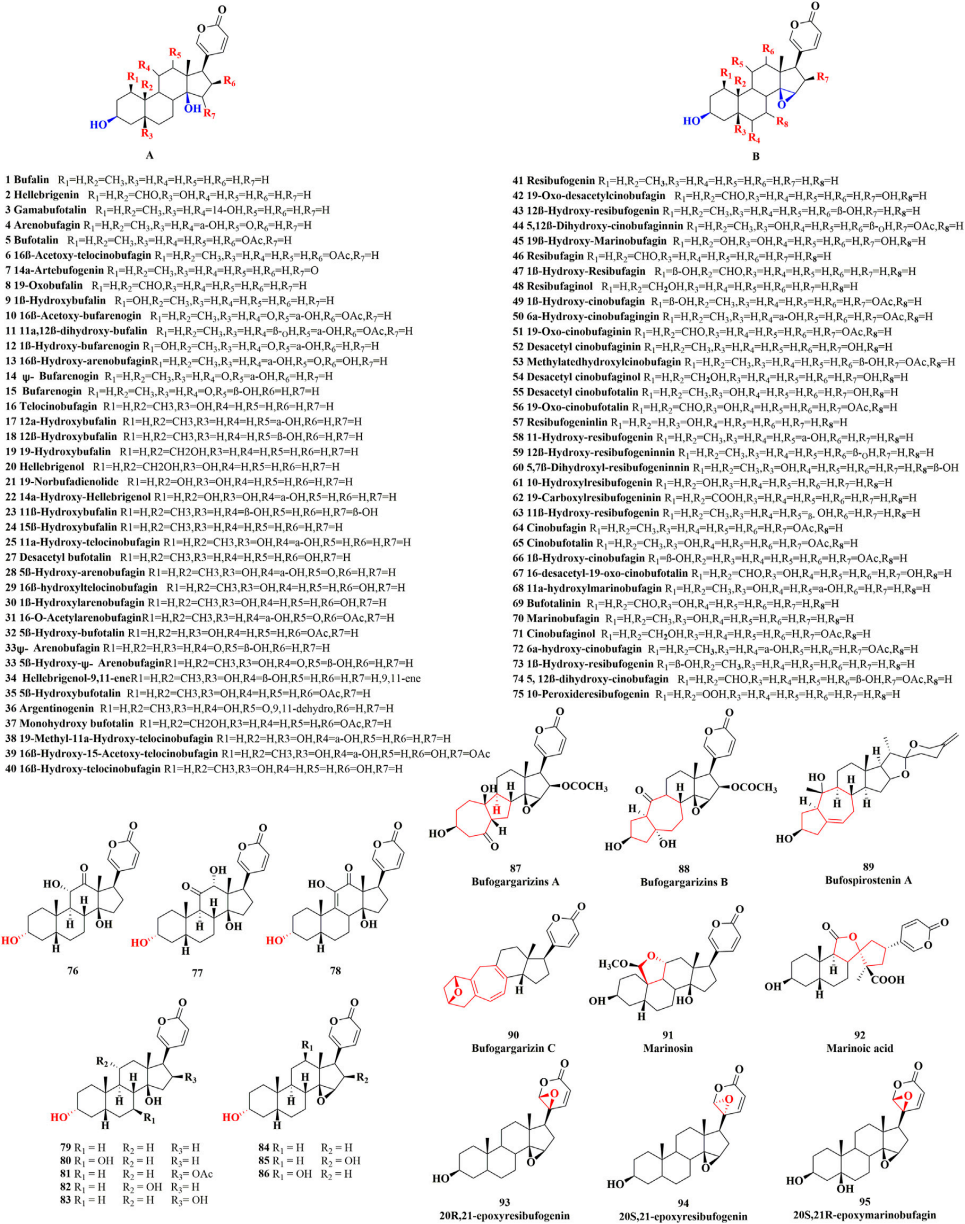
The chemical structures of reported free bufadienolide.

Unsubstituted *α* or *β* hydroxyl groups at the C-3 position are the distinguishing features of free bufadienolide. Depending on whether the C-14 position is substituted by a *β*-OH or the C-14, 15 positions form a *β*-epoxy structure, free bufadienolide could be divided into two categories (Group 1 and Group 2 in Figure 1B), which could be further classified into five sub-categories based on the contained function groups (hydroxy, aldehyde, carbonyl, etc.) (Figure 1B). It was reported that at least 75 free bufadienolides (***1–75***) have been identified (Li et al., 2015; Rodríguez et al., 2017; Wei et al., 2019; Zhan et al., 2020), whose chemical structures were shown in Figure 2.

In recent years, the finding of free bufadienolide with 3*α*-OH configuration in toad bile, the epimer of the ubiquitously disseminated free bufadienolides with 3*β*-OH configuration, has merited our attention (Zou et al., 2018). Generally, free bufadienolide with 3*α*-OH configuration originated from microbial transformation or animal metabolism (Zhang et al., 2011; Bhatti and Khera 2012; Hegazy et al., 2015; Wei et al., 2022), whose natural occurrence is quite rare. Our group isolated and identified eleven free bufadienolides (***76–86***) with 3*α*-OH configuration from the bile of *Bufo gargarizans* (unpublished data) (Figure 2).

With the development of chromatography technology and unremitting efforts of researchers (Guillarme et al., 2010), novel structures or new backbone type of free bufadienolide have continued to be discovered. Specifically, six free bufadienolides of new backbone type (Matsukawa et al., 1996; Matsukawa et al., 1997; Tian et al., 2010; Tian et al., 2017), namely Bufogargarizins A, Bufogargarizins B, Bufogargarizins C, Bufospirostenin A, Marinosin and Marinosin acid (***87–92***), were discovered and isolated from *Bufo sinensis* and Cane toad. Meanwhile, a rare series of 20, 21-epoxybufenolides (Nogawa et al., 2001; Kamano et al., 2002; Chen et al., 2015; Chen et al., 2018), namely 20S, 21-epoxyresibufogenin, 20R, 21-epoxyresibufogenin and 20S, 21R-epoxymarinobufagin (***93–95***), were discovered and isolated from the Brazilian toad *Rhinella schneideri* (Figure 2).

### Conjugated bufadienolide

**FIGURE 3:**
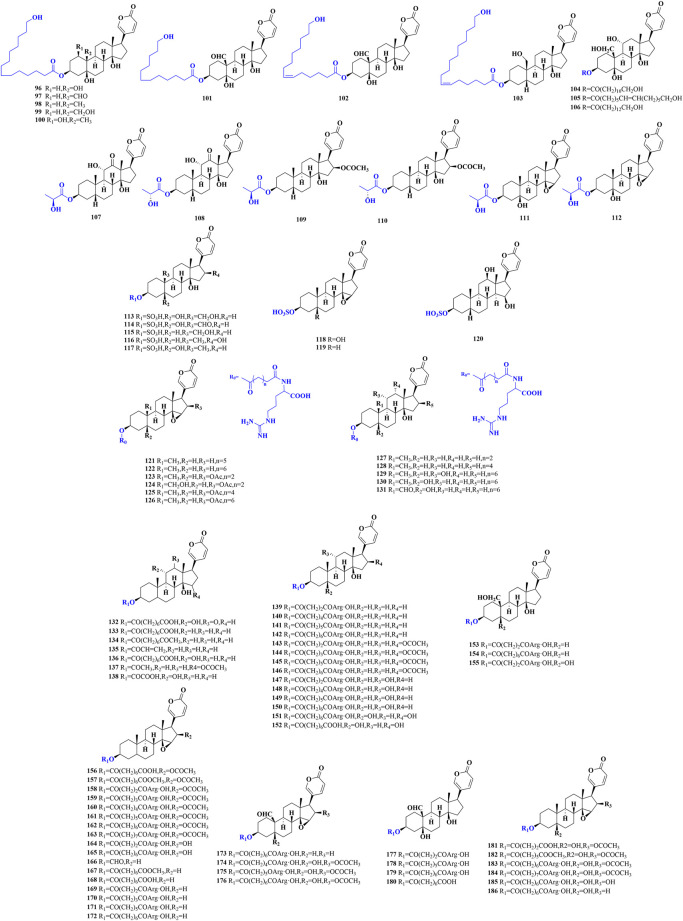
The chemical structures of reported conjugated bufadienolide.

The substitution of C-3 hydroxyl group is the defining property of conjugated bufadienolide, which often appears in a form of ester (Figure 3), including sulfonic acid, amino acid, fatty acid and arginine diacyl chain esterification (Gao et al., 2010; Zhou et al., 2017; Zhou et al., 2021).

Eight conjugated bufadienolides that C-3 position esterized with fatty acid from the egg of *Bufo bufo gargarizans* were isolated and identified by Zhang et al. (***96–103***) (Zhang et al., 2016). Besides, three unusual conjugated bufadienolides were separated from the ovaries of *Bufo marinus* (***104–106***) (Matsukawa et al., 1998). Zhou et al. isolated and identified three pairs of epimers in which the C-3 hydroxyl groups were substituted by lactic acid from the egg of *Bufo bufo gargarizans* (***107–112***) (Zhou et al., 2017). It was the first time to reveal the racemization of lactate in amphibians.

Gao et al. isolated and identified five sulfonated bufadienolides from the venom of *Bufo Melanosticus* by the joint use of LC-DAD-MS^n^ and LC-SPE-NMR (***113–117***) (Gao et al., 2010). From the bile of the Cane toad, three sulfonated bufadienolides (***118–120***) were also characterized by Lee et al. (Lee et al., 1994). The sulfonated bufadienolide exhibited better water solubility compared to the corresponding free bufadienolide. Bufadienolide sulfonation may be one of the metabolism and detoxification mechanisms of bufadienolide in animals and it may also indicate that the toxicity of sulfonated bufadienolide could be significantly reduced.

In addition to fatty acid and sulfonic acid conjugated bufadienolide, arginine diacyl ester is the predominant form of conjugated bufadienolide. It is characterized by the esterification of C-3 hydroxyl group with an arginine diacyl group, and the variable length of the arginine diacyl chain leads to the structure diversity of this kind of conjugated bufadienolide. Zhou et al. identified a series of arginine diacyl chain conjugated bufadienolides from *Bufo Bufoni* by UPLC-QQQ-MS analysis (***121–131***) (Zhou et al., 2015). Furthermore, at least 55 arginine diacyl chain conjugated bufadienolides have been isolated and identified from Chansu (***132–186***) (Gao et al., 2011; Wei et al., 2019; Zhan et al., 2020; Li et al., 2021), and their chemical structures were shown in Figure 3.

It was worth mentioning that Argentine researchers discovered a high concentration of arginine diacyl chain conjugated bufadienolides (***187–276***) in the venom of Argentine toad by MALDI-MS analysis (Table 1) (Petroselli et al., 2018). Furthermore, the characteristic fragment ions indicated that there may still exist numerous arginine diacyl chain conjugated bufadienolides polymerized as dimers or multimers remained to be discovered.

**TABLE 1:** Conjugated bufadienolide detected in Argentine Toad venom by MALDI-MS.

No.	*m/z*	Adduct	Tentative structure assigned
**187**	657	[M + H]^+^	3-(*N*-glutaryl argininyl)-bufalin
**188**	669	[M + H]^+^	3(*N*-adipoyl argininyl)-resibufogenin
**189**	671	[M + H]^+^	3-(*N*-adipoyl argininyl)-bufalin
**190**	683	[M + H]^+^	3-(*N*-pimeloyl argininyl)-resibufogenin
**191**	685	[M + H]^+^	3-(*N*-pimeloyl argininyl)-bufalin/3-(N-adipoyl argininyl)-marinobufagin
**192**	691	[M + Na]^+^	3-(*N*-adipoyl argininyl)-resibufogenin
**193**	693	[M + Na]^+^	3-(*N*-adipoyl argininyl)-bufalin
**194**	696	[M + Na]+	3-(*N*-glutaryl argininyl)-telocinobufagin
**195**	699	[M + H]^+^	3-(*N*-suberoyl argininyl)-bufalin (bufalitoxin)/3-(*N*-pimeloyl argininyl)-marinobufagin)/3-(*N*-adipoyl)-bufotalinin
**196**	701	[M + H]^+^	3-(*N*-pimeloyl argininyl)-telocinobufagin, 3-(*N*-adipoyl argininyl)-bufarenogin or arenobufagin or hellebrigenin
**197**	707	[M + Na]^+^	3-(*N*-pimeloyl argininyl)-bufalin/3-(*N*-adipoyl argininyl)-marinobufagin
**198**	710	[M + Na]^+^	3-(*N*-adipoyl argininyl)-telocinobufagin
**199**	713	[M + H]^+^	3-(*N*-azelayl argininyl)-bufalin/3-(*N*-suberoyl argininyl)-marinobufagin (marinobufotoxin)/3-(*N*-pimeloyl argininyl)-bufotalinin
**200**	715	[M + H]^+^	3-(*N*-suberoyl argininyl)-telocinobufagin (telocinobufatoxin), 3-(*N*-pimeloyl argininyl)-bufarenogin or arenobufagin or hellebrigenin
**201**	721	[M + Na]^+^	3-(*N*-suberoyl argininyl)-bufalin (bufalitoxin)/3-(*N*-pimeloyl argininyl)- marinobufagin/)/3-(*N*-adipoyl)-bufotalinin
**202**	724	[M + Na]^+^	3-(*N*-pimeloyl argininyl)-telocinobufagin, 3-(*N*-adipoyl argininyl)- bufarenogin or arenobufagin or hellebrigenin
**203**	727	[M + H]^+^	3-(*N*-sebacyl argininyl)-bufalin/3-(*N*-azelayl argininyl)-marinobufagin/3-(*N*-suberoyl argininyl)-bufotalinin
**204**	729	[M + H]^+^	3-(*N*-adipoyl argininyl)-bufotalin/3-(*N*-suberoyl argininyl)-bufarenogin or arenobufagin or hellebrigenin
**205**	736	[M + Na]^+^	3-(*N*-azelayl argininyl)-bufalin/3-(*N*-suberoyl argininyl)-marinobufagin (marinobufotoxin)/3-(*N*-pimeloyl argininyl)-bufotalinin
**206**	738	[M + Na]^+^	3-(*N*-suberoyl argininyl)-telocinobufagin (telocinobufatoxin)/3-(*N*-pimeloyl argininyl)-bufarenogin or arenobufagin or hellebrigenin
**207**	752	[M + Na]^+^	3-(*N*-suberoyl argininyl)-bufarenogin or arenobufagin or hellebrigenin
**208**	757	[M + H]^+^	3-(*N*-suberoyl argininyl)-bufotalin (bufotoxin)/3-(*N*-undecadienoyl argininyl)-telocinobufagin/3-(*N*-sebacyl argininyl)-bufarenogin or arenobufagin or hellebrigenin
**209**	1339	[M + H]^+^	3-(*N*- adipoyl argininyl)-resibufogenin dimer
**210**	1355	[M + H]^+^	[3-(*N*- adipoyl argininyl)-resibufogenin]-[3-(*N*- adipoyl argininyl)-marinobufagin] dimer
**211**	1371	[M + H]^+^	[3-(*N*- adipoyl argininyl)-marinobufagin] dimer
**212**	1367	[M + H]^+^	[3-(*N*- adipoyl argininyl)-resibufogenin]-[3-(*N*-suberoyl argininyl)-bufalin] dimer
**213**	1369	[M + H]^+^	[3-(*N*- adipoyl argininyl)-resibufogenin]-[3-(*N*- adipoyl)-bufotalinin] dimer
**214**	1369	[M + H]^+^	3-(*N*- adipoyl argininyl)-resibufogenin and 3-(*N*- adipoyl argininyl)-bufarenogin or arenobufagin or hellebrigenin dimer
**215**	1367	[M + H]^+^	[3-(*N*- adipoyl argininyl)-bufalin]-[3-(*N*-suberoyl argininyl)-bufalin] dimer
**216**	1369	[M + H]^+^	[3-(*N*- adipoyl argininyl)-bufalin]-[3-(*N*- adipoyl)-bufotalinin] dimer
**217**	1383	[M + H]^+^	[3-(*N*- adipoyl argininyl)-resibufogenin]-[ 3-(*N*-suberoyl argininyl)-telocinobufagin] dimer
**218**	1383	[M + H]^+^	3-(*N*- adipoyl argininyl)-resibufogenin and 3-(*N*-pimeloyl argininyl)-bufarenogin or arenobufagin or hellebrigenin dimer
**219**	1383	[M + H]^+^	[3-(*N*- adipoyl argininyl)-bufalin]-[3-(*N*-suberoyl argininyl)-telocinobufagin] dimer
**220**	1383	[M + H]^+^	3-(*N*- adipoyl argininyl)-bufalin and 3-(*N*-pimeloyl argininyl)-bufarenogin or arenobufagin or hellebrigenin dimer
**221**	1383	[M + H]^+^	[3-(*N*-pimeloyl argininyl)-telocinobufagin]-[3-(*N*- pimeloyl argininyl)-bufalin] dimer
**222**	1385	[M + H]^+^	[3-(*N*-pimeloyl argininyl)-telocinobufagin]-[3-(*N*- adipoyl argininyl)-marinobufagin] dimer
**223**	1383	[M + H]^+^	3-(*N*- adipoyl argininyl)-bufarenogin or arenobufagin or hellebrigenin and 3-(*N*- pimeloyl argininyl)-bufalin dimer
**224**	1385	[M + H]^+^	3-(*N*- adipoyl argininyl)-bufarenogin or arenobufagin or hellebrigenin and 3-(*N*- adipoyl argininyl)-marinobufagin dimer
**225**	1381	[M + H]^+^	[3-(*N*-suberoyl argininyl)-bufalin]-[3-(*N*- pimeloyl argininyl)-bufalin] dimer
**226**	1383	[M + H]^+^	[3-(*N*-suberoyl argininyl)-bufalin]-[3-(*N*- adipoyl argininyl)-marinobufagin] dimer
**227**	1383	[M + H]^+^	[3-(*N*- pimeloyl argininyl)-marinobufagin]-[3-(*N*- pimeloyl argininyl)-bufalin] dimer
**228**	1385	[M + H]^+^	[3-(*N*- pimeloyl argininyl)-marinobufagin]-[3-(*N*- adipoyl argininyl)-marinobufagin] dimer
**229**	1383	[M + H]^+^	[3-(*N*- adipoyl)-bufotalinin]-[3-(*N*- pimeloyl argininyl)-bufalin] dimer
**230**	1385	[M + H]^+^	[3-(*N*- adipoyl)-bufotalinin]-[3-(*N*- adipoyl argininyl)-marinobufagin] dimer
**231**	1397	[M + H]^+^	[3-(*N*- adipoyl argininyl)-resibufogenin]-[ 3-(*N*- adipoyl argininyl)-bufotalin] dimer
**232**	1397	[M + H]^+^	3-(*N*- adipoyl argininyl)-resibufogenin and 3-(*N*- suberoyl argininyl)-bufarenogin or arenobufagin or hellebrigenin dimer
**233**	1399	[M + H]^+^	[3-(*N*- adipoyl argininyl)-bufalin]-[3-(*N*- adipoyl argininyl)-bufotalin] dimer
**234**	1397	[M + H]^+^	3-(*N*- adipoyl argininyl)-bufalin and 3-(*N*- suberoyl argininyl)-bufarenogin or arenobufagin or hellebrigenin dimer
**235**	1397	[M + H]^+^	[3-(*N*-suberoyl argininyl)-telocinobufagin]-[3-(*N*- pimeloyl argininyl)-bufalin] dimer
**236**	1399	[M + H]^+^	[3-(*N*-suberoyl argininyl)-telocinobufagin]-[3-(*N*- adipoyl argininyl)-marinobufagin] dimer
**237**	1397	[M + H]^+^	3-(*N*-pimeloyl argininyl)-bufarenogin or arenobufagin or hellebrigenin and 3-(*N*- pimeloyl argininyl)-bufalin dimer
**238**	1399	[M + H]^+^	3-(*N*-pimeloyl argininyl)-bufarenogin or arenobufagin or hellebrigenin and 3-(*N*- adipoyl argininyl)-marinobufagin dimer
**239**	1395	[M + H]^+^	[3-(*N*-suberoyl argininyl)-bufalin] dimer
**240**	1397	[M + H]^+^	[3-(*N*- adipoyl)-bufotalinin] dimer
**241**	1397	[M + H]^+^	[3-(*N*- pimeloyl argininyl)-marinobufagin]dimer
**242**	1411	[M + H]^+^	[3-(*N*- adipoyl argininyl)-bufotalin]-[3-(*N*- pimeloyl argininyl)-bufalin] dimer
**243**	1413	[M + H]^+^	[3-(*N*- adipoyl argininyl)-bufotalin]-[3-(*N*- adipoyl argininyl)-marinobufagin] dimer
**244**	1411	[M + H]^+^	3-(*N*- suberoyl argininyl)-bufarenogin or arenobufagin or hellebrigenin and 3-(*N*- pimeloyl argininyl)-bufalin dimer
**245**	1413	[M + H]^+^	3-(*N*- suberoyl argininyl)-bufarenogin or arenobufagin or hellebrigenin and 3-(*N*- adipoyl argininyl)-marinobufagin dimer
**246**	1411	[M + H]^+^	[3-(*N*- suberoyl argininyl)-marinobufagin]-[3-(*N*-suberoyl argininyl)-bufalin] dimer
**247**	1411	[M + H]^+^	[3-(*N*- suberoyl argininyl)-marinobufagin]-[3-(*N*- pimeloyl argininyl)-marinobufagin] dimer
**248**	1411	[M + H]^+^	[3-(*N*- suberoyl argininyl)-marinobufagin]-[3-(*N*- adipoyl)-bufotalinin] dimer
**249**	1411	[M + H]^+^	3-(*N*-pimeloyl argininyl)-bufotalinin and 3-(*N*-suberoyl argininyl)-bufalin dimer
**250**	1411	[M + H]^+^	3-(*N*-pimeloyl argininyl)-bufotalinin and 3-(*N*- pimeloyl argininyl)-marinobufagin dimer
**251**	1411	[M + H]^+^	3-(*N*-pimeloyl argininyl)-bufotalinin and 3-(*N*- adipoyl)-bufotalinin dimer
**252**	1427	[M + H]^+^	3-(*N*-suberoyl argininyl)-telocinobufagin dimer
**253**	1427	[M + H]^+^	3-(*N*-suberoyl argininyl)-telocinobufagin and 3-(*N*-pimeloyl argininyl)-bufarenogin or arenobufagin or hellebrigenin dimer
**254**	1427	[M + H]^+^	3-(*N*-pimeloyl argininyl)-bufarenogin or arenobufagin or hellebrigenin dimer
**255**	1427	[M + H]^+^	3-(*N*-suberoyl argininyl)-telocinobufagin and 3-(*N*- suberoyl argininyl)-marinobufagin dimer
**256**	1427	[M + H]^+^	3-(*N*-suberoyl argininyl)-telocinobufagin and 3-(*N*-pimeloyl argininyl)-bufotalinin dimer
**257**	1427	[M + H]^+^	3-(*N*-pimeloyl argininyl)-bufarenogin or arenobufagin or hellebrigenin and 3-(*N*- suberoyl argininyl)-marinobufagin dimer
**258**	1427	[M + H]^+^	3-(*N*-pimeloyl argininyl)-bufarenogin or arenobufagin or hellebrigenin and 3-(*N*-pimeloyl argininyl)-bufotalinin dimer
**259**	1427	[M + H]^+^	3-(*N*- suberoyl argininyl)-bufarenogin or arenobufagin or hellebrigenin and 3-(*N*-pimeloyl argininyl)-telocinobufagin dimer
**260**	1427	[M + H]^+^	3-(*N*- suberoyl argininyl)-bufarenogin or arenobufagin or hellebrigenin and 3-(*N*- adipoyl argininyl)-bufarenogin or arenobufagin or hellebrigenin dimer
**261**	1427	[M + H]^+^	[3-(*N*- adipoyl argininyl)-bufotalin]-[ 3-(*N*-pimeloyl argininyl)-telocinobufagin] dimer
**262**	1427	[M + H]^+^	3-(*N*- adipoyl argininyl)-bufotalin and 3-(*N*- adipoyl argininyl)-bufarenogin or arenobufagin or hellebrigenin dimer
**263**	1425	[M + H]^+^	3-(*N*- suberoyl argininyl)-bufarenogin or arenobufagin or hellebrigenin and 3-(*N*-suberoyl argininyl)-bufalin dimer
**264**	1427	[M + H]^+^	3-(*N*- suberoyl argininyl)-bufarenogin or arenobufagin or hellebrigenin and 3-(*N*- pimeloyl argininyl)-marinobufagin dimer
**265**	1427	[M + H]^+^	3-(*N*- suberoyl argininyl)-bufarenogin or arenobufagin or hellebrigenin and 3-(*N*- adipoyl)-bufotalinin dimer
**266**	1425	[M + H]^+^	[3-(*N*- adipoyl argininyl)-bufotalin]-[3-(*N*-suberoyl argininyl)-bufalin] dimer
**267**	1427	[M + H]^+^	[3-(*N*- adipoyl argininyl)-bufotalin]-[3-(*N*- pimeloyl argininyl)-marinobufagin] dimer
**268**	1427	[M + H]^+^	[3-(*N*- adipoyl argininyl)-bufotalin]-[3-(*N*- adipoyl)-bufotalinin] dimer
**269**	1439	[M + H]^+^	[3-(*N*- adipoyl argininyl)-bufotalin ]-[3-(*N*- suberoyl argininyl)-marinobufagin] dimer
**270**	1441	[M + H]^+^	[3-(*N*- adipoyl argininyl)-bufotalin]-[3-(*N*-pimeloyl argininyl)-bufotalinin] dimer
**271**	1439	[M + H]^+^	3-(*N*- suberoyl argininyl)-bufarenogin or arenobufagin or hellebrigenin and 3-(*N*- suberoyl argininyl)-marinobufagin dimer
**272**	1441	[M + H]^+^	3-(*N*- suberoyl argininyl)-bufarenogin or arenobufagin or hellebrigenin and 3-(*N*-pimeloyl argininyl)-bufotalinin dimer
**273**	1441	[M + H]^+^	[3-(*N*- adipoyl argininyl)-bufotalin]-[3-(*N*-suberoyl argininyl)-telocinobufagin] dimer
**274**	1441	[M + H]^+^	[3-(*N*- adipoyl argininyl)-bufotalin and 3-(*N*-pimeloyl argininyl)-bufarenogin or arenobufagin or hellebrigenin dimer
**275**	1441	[M + H]^+^	3-(*N*- suberoyl argininyl)-bufarenogin or arenobufagin or hellebrigenin and 3-(*N*-suberoyl argininyl)-telocinobufagin dimer
**276**	1441	[M + H]^+^	3-(*N*- suberoyl argininyl)-bufarenogin or arenobufagin or hellebrigenin and 3-(*N*-pimeloyl argininyl)-bufarenogin or arenobufagin or hellebrigenin dimer

## Bufadienolide MS fragmentation principles

**FIGURE 4:**
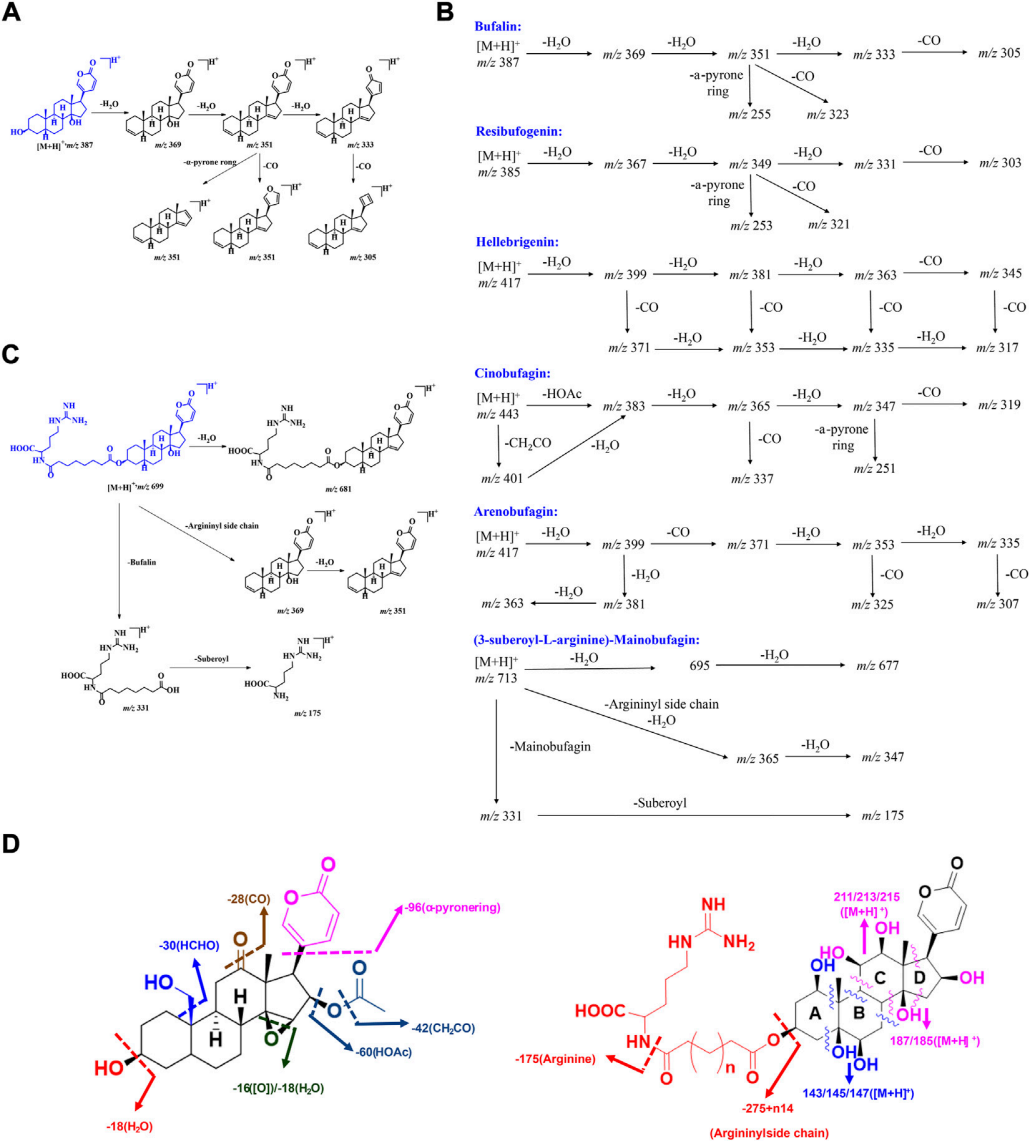
The MS fragmentation principles of bufadienolide: **(A**–**C)** The typical fragmentation pathways of free and conjugated bufadienolide; **(D)** The summarized fragmentation patterns of free and conjugated bufadienolide.

In the realm of natural products, the ion response of bufadienolide is more sensitive in the positive ion mode of electrospray ionization (ESI) than in the negative ion mode. The MS/MS spectra of bufadienolide could be divided into two regions: the steroid core region is located at *m/z* 50-250, while the functional group region spans *m/z* 250-500. In the functional group region, it is common to observe the fragment ions resulting from the loss of a series of neutral molecules, such as H_2_O and CO, which correspond to the fragmentation of certain functional groups including hydroxyl, aldehyde, and carbonyl et al. For the steroidal core region, the fragment ions are acquired primarily by the Retro-Diels-Alder (RDA) reaction, induced cleavage, and hydrogenation rearrangement (Tureček and Hanuš 1984; Silva and Lopes 2015). Based on the MS/MS fragmentation of bufadienoldide standards and literature reports (Han et al., 2016; Meng et al., 2016; Han et al., 2017), the general MS/MS fragmentation principles for bufadienolide are summarized as follows (Figure 4).

### Bufalin and its polyhydroxy derivatives

Bufalin produced an obvious [M + H]^+^ ion at *m/z* 387.2515. After Collision-Induced Dissociation (CID), a series of MS/MS ions, including [M + H-18]^+^ (*m/z*, 369.2414), [M + H-36]^+^ (*m/z*, 351.2309), [M + H-54]^+^ (*m/z*, 333.2214) and [M + H-36-96]^+^ (*m/z*, 255.2100), were produced, which were evoked by successive losses of H_2_O and CO. Meanwhile, a strong intensity ion at *m/z* 255.2100 (a neutral loss of 96 Da) was observed, and it was initiated by the elimination of α-pyrone ring at C-17 position. The possible fragmentation pathway of bufalin is shown in Figures 4A,B. The polyhydroxy derivatives of bufalin showed similar fragmentation patterns to bufalin: successive losses of H_2_O and CO, which are evoked by the elimination of specific functional groups. They are followed by a neutral loss of 96 Da triggered by the elimination of α-pyrone ring at the C-17 position.

### Resibufogenin and its polyhydroxy derivatives

The MS/MS spectrum of resibufogenin exhibited very similar patterns to those of bufalin. Obvious fragment ions, including [M + H-18]^+^ (*m/z*, 367.2263), [M + H-36]^+^ (*m/z*, 349.2155), [M + H-18-28]^+^ (*m/z*, 339.2310) and [M + H-36-96]^+^ (*m/z*, 253.1948), were abundant in the MS/MS spectrum. The above ions were given by successive losses of H_2_O and CO by elimination of hydroxyl groups, C-14/15 epoxy ring, and α-pyrone ring fragmentation (Figure 4B). Their MS/MS fragment ions generally could be correlated with the number of equipped hydroxyl groups. The whole hydroxyl groups, including C-14/15 epoxy ring, tended to be eliminated to give a stable fragment ion.

### Bufadienolide with aldehyde substituent

This group of bufadienolide is characterized by an aldehyde group positioned at C-10. Hellebrigenin is a typical bufadienolide with acetoxyl substituent. It produced an obvious [M + H]^+^ ion at *m/z* 417.2257. After CID, hellebrigenin gave a series of MS/MS ions including [M + H-18]^+^ (*m/z*, 399.2165), [M + H-36]^+^ (*m/z*, 381.2068), [M + H-54]^+^ (*m/z*, 363.1952), [M + H-18-28]^+^ (*m/z*, 371.2221), [M + H-36-28]^+^ (*m/z*, 353.2109), [M + H-54-28]^+^ (*m/z*, 335.1996) and [M + H-54-28-96]^+^ (*m/z*, 239.1793). Obviously, a series of MS/MS ions characterized by the loss of 28 (CO) were clearly more abundant for bufadienolide with aldehyde substituent than other types of bufadienolide. These ions presumably resulted from the elimination of CO from aldehyde group positioned at C-10 (Figure 4B).

### Bufadienolide with acetoxyl substituent

Bufadienolide with acetoxyl substituent is usually equipped with an acetoxyl group at the C-16 position, giving a characteristic fragmentation pattern. The obvious and abundant MS/MS fragment ions of Cinobufalin, a typical bufadienolide with acetoxyl substituent, usually included [M + H-42]^+^ (*m/z*, 417.2273), [M + H-60]^+^ (*m/z*, 399.2175), [M + H-60-18]^+^ (*m/z*, 381.2063), [M + H-60-36]^+^ (*m/z*, 363.1952), [M + H-60-54]^+^ (*m/z*, 345.1855). In general, the specific ions, namely [M + H-42]^+^ and [M + H-60]^+^, are the characteristic ions for this kind of bufadienolide. They are produced by the elimination of acetoxyl group to trigger the initial loss of 42 (CH_2_CO) or 60 (HOAc) Da (Figure 4B). The remaining ions are produced by consecutive losses of H_2_O and CO evoked by the specific functional group fragmentation, as with the above three groups of bufadienolide.

### Bufadienolide with carbonyl substituent

This group of bufadienolide contains a carbonyl group at the C-11 or C-12 position. A typical bufadienolide belonging to this group is arenobufagin, which contains a carbonyl group at the C-11 position. The MS/MS fragment ions of arenobufagin typically consisted of [M + H-18]^+^ (*m/z*, 399.2150), [M + H-36]^+^ (*m/z*, 381.2060), [M + H-54]^+^ (*m/z*, 363.1954), [M + H-18-28]^+^ (*m/z*, 371.2220), [M + H-36-28]^+^ (*m/z*, 353.2115), [M + H-54-28]^+^ (*m/z*, 335.2003) and [M + H-54-28-96]^+^ (*m/z*, 239.1797). Characteristically, the existence of a carbonyl group leads to the neutral loss of 28 Da (CO) to generate the corresponding diagnostic ions (Figure 4B). Importantly, the relative intensity of diagnostic ions, in terms of the ions at m/z 399 generated by the elimination of C-3 hydroxyl group and its subsequent CO eliminated ions, could be utilized to distinguish between isomers of bufadienolides with the carbonyl group substituted at C-11 and C-12 position.

### Conjugated bufadienolide (substituted with arginine diacyl chain)

For conjugated bufadienolide, strong arginine diacyl chain ions and a series of fragment ions formed by the subsequent fragmentation of theses diacyl chains were given due to the strong ability of N to stabilize the positive charge. Meanwhile, the bufadienolide part, giving rather weak ion peaks in MS/MS spectrum, was eliminated as a neutral molecular (Figures 4B,C). (3-suberoyl-L-arginine)-Mainobufagin is a typical conjugated bufadienolide with an obvious [M + H]^+^ ion at *m/z* 713.4089 in its MS spectrum. After CID, a series of MS/MS fragment ions, including [M + H-18]^+^ (*m/z*, 695.3990), [M + H-36]^+^ (*m/z*, 677.3921), [M + H-382]^+^ (*m/z*, 331.1958), and [M + H-382-156]^+^ (*m/z*, 175.1188), were produced. When employing negative ion mode and increasing the induced collision voltage in MS/MS, the ion intensity of bufadienolide part could be significantly increased. However, it is still rather weak compared to that of arginine diacyl chain fragmentation.

MS fragmentation principles of bufadienolide could be primarily and briefly summarized as follows: 1) Bufadienolide containing solely hydroxyl groups can be detected by consecutive losses of H_2_O and CO, and the amount of hydroxyl groups is generally associated with the resulting ion profile. Unfortunately, determination the hydroxyl substitution location solely based on MS/MS data is difficult or impossible. Meanwhile, the achievement of the 14-OH and 14/15-epoxy ring distinguishment is also impossible just by the MS/MS profile; 2) CO elimination is informative. It may indicate the presence of a C-10 aldehyde group and a carbonyl group at C-11 or C-12 position; 3) The specific loss of 30 Da (HCHO) indicates the presence of a C-19 hydroxyl group; 4) The characteristic loss of 42 Da (CH_2_CO) and 60 Da (HOAc) indicates the presence of an acetoxyl group at the C-16 position; 5) Strong arginine diacyl chain ion peaks, accompanied by a series of fragment ions formed by the subsequent elimination of these chains, indicate the presence of arginine diacyl chains for conjugated bufadienolide (Figure 4D).

## Anti-inflammatory efficacy

On account of the molecular level regulatory ability and specific Na^+^/K^+^ ATPase inhibitory activity, bufadienolide exhibited excellent bioactivities, including cardiotonic, anti-inflammatory, anti-tumor, immunoregulatory, NO production, renal sodium excretion and blood pressure stimulating (Prassas and Diamandis 2008; de Vasconcelos et al., 2011; Gao et al., 2011; Wen et al., 2014). Of the whole activities defined for bufadienolide, anti-inflammatory activity may be one of the most promising and interesting research topics.

### Specific inhibition of Na^+^/K^+^-ATPase

**FIGURE 5:**
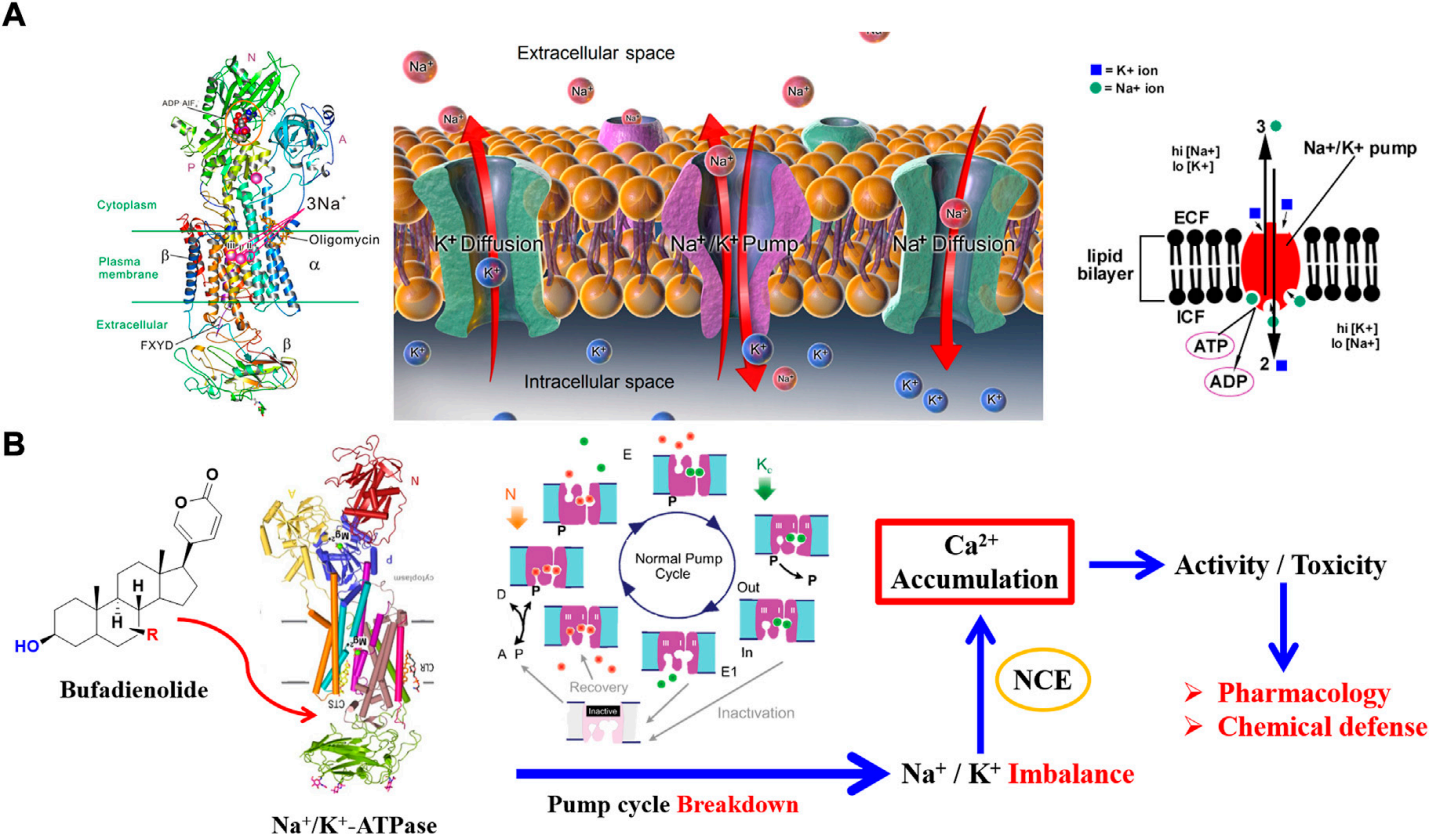
Specific inhibition of bufadienolide against Na^+^/K^+^ ATPase: **(A)** The crystal structure and function of Na^+^/K^+^ ATPase; **(B)** The primary act mechanism of bufadienolide against Na^+^/K^+^ ATPase.

Na^+^/K^+^ ATPase was discovered in 1957 by the Danish scientist Jens Christian Skou, who was awarded a Nobel Prize for this milestone discovery in 1997 (Boyer et al., 1997). The predominant function of Na^+^/K^+^ ATPase is to transport K^+^ and Na^+^ in a manner of reverse gradient using the energy fueled by ATP hydrolysis. For a detailed transport procedure, two K^+^ are transported into the cell, while three Na^+^ are transferred out of the cell. By cooperating with ion free diffusion, cell resting potential could be maintained to a state that electrochemical gradient keeps equal on both sides of the cell membrane (Figure 5A). This discovery marked an important step forward in the understanding of how ions get into and out of cells (Jørgensen 1982; Nyblom et al., 2013).

Structurally, three subunits (α subunit, β subunit, and FXYD subunit) compose the structure of Na^+^/K^+^ ATPase (Figure 5A). It is the distribution and expression difference of the three subunits in various tissue that achieves the functional diversity of Na^+^/K^+^ ATPase (Morth et al., 2007; Kanai et al., 2013). There exist four subtypes of *α* subunit, namely *α1, α2, α3,* and *α4* subunit, that have been discovered in human beings (Sweadner 1989). Of the four subtypes of *α* subunit, *α1* subtype is predominant and is found in almost all the body cells. In contrast, *α2* subtype, *α3* subtype, and *α4* subtype mainly distribute in cardiomyocytes, nerve cells, and testis tissue, respectively.

The most attractive point of bufadienolide is cardiotonic activity, which is triggered by the specific inhibition of Na^+^/K^+^ ATPase. For a detailed action mechanism, the specific inhibition of Na^+^/K^+^ ATPase leads to an increase of intracellular Na^+^ concentration and a decrease of the K^+^ concentration. The above ions concentration change will activate Na^+^/Ca^2+^ exchanger on the sarcoplasmic reticulum to regulate the Ca^2+^ concentration for a balanced cell electrochemical gradient. Eventually, a positive inotropic activity is achieved *via* promoting the contraction of troponin in cardiomyocytes triggered by the upregulated Ca^2+^ concentration (Ogawa et al., 2009; Laursen et al., 2015). However, serious toxicity would be obtained if the intracellular calcium ion is excessively accumulated (Figure 5). As mentioned above, both the cell membrane of the whole tissue in the body almost distributes the *α1* subtype of Na^+^/K^+^ ATPase, which will result in a narrow therapeutic safety window. Consequently, the therapeutic dose has reached the level of 60% of the toxic dose due to its double-edged sword property (Huang et al., 2019). Thus, it is emergency to discover Na^+^/K^+^ ATPase inhibitors with high selectivity that can regulate the activity of specific subtypes of Na^+^/K^+^ ATPase present in targeted tissues.

### Anti-inflammatory activity

Generally, anti-inflammatory activity is closely linked with anticancer activity. Bufadienolide has been comprehensively studied for its anticancer activity in diverse types of cancer, such as breast cancer, lung carcinoma, human leukemia, gastrointestinal, hepatoma et al. The underlying anticancer mechanisms were always linked with anti-inflammatory pathway (Prassas and Diamandis 2008; Gao et al., 2011; Deng et al., 2021).

It was revealed that chronic inflammation could result in tissue damage accompanied by an increased risk of cancer development. Generally, the microenvironment with inflammation is the prerequisite for cancer progression. Cancer tissues would frequently produce inflammation without the occurrence of precancerous inflammation (Coussens and Werb 2002). For cancer-involved inflammation, the expression level of numerous inflammatory products is dramatically affected by a predominant transcription factor namely NF-κB (Liu et al., 2017). Consequently, inflammatory diseases, such as rheumatoid arthritis, could be treated by some anticancer drugs. In turn, it provides a promising approach for cancer prevention and therapy by inflammation elimination (Todoric et al., 2016).

Bufadienolide has been long reported for anti-inflammatory activity, especially for cancer-involved inflammation (Gao et al., 2011; Qi et al., 2014; Rong et al., 2014). Ye found that bufalin exhibited anti-inflammatory activity by blocking TNF (tumor necrosis factor), which was mediated *via* the NF-κB nuclear translocation. Jiang found that NF-κB expression and phosphorylation could be suppressed by bufalin (Jiang et al., 2010), which supported the previous conclusion. An investigation led by Chen revealed that phosphoinisitide-3-kinase (PI3K) and phosphorylation of AKT could be attenuated by bufalin in the SK-Hep1 cell line. Importantly, this attenuation was triggered by the decreased expression level of NF-κB (Chen et al., 2015). Furthermore, Dong demonstrated that the expression of NF-κB p65 protein could be dramatically suppressed by cinobufagin in the HepG2 cell line (Dong et al., 2010).

Meanwhile, NO, a key inflammatory factor, was found to be a predominant signaling molecule regulated by bufadienolide in the tumorigenesis process. Bhuiyan further confirmed the NO regulatory ability of bufalin (Bhuiyan et al., 2003). Their work also showed an interesting phenomenon: NO production was significantly up-regulated by a low concentration of bufalin, while bufalin with a high concentration gave the opposite result. In another study led by Kim *via* the BV2 microglial cell line, it was found that the expression of two key inflammatory-related enzymes, namely iNOS and COX-2, was significantly inhibited by Chansu. At the same time, it also suppressed the production of NO and PGE2 (Kim et al., 2008). Wang further discovered the powerful anti-inflammatory activity of Huachansu. It was found that TNF-α mediated NF-κB and COX-2 activation were inhibited, and the expression levels of two pro-inflammatory cytokines, namely IL-6 and IL-8, were decreased by bufadienolide in Huchansu (Wang et al., 2019).

## Structure modification

Despite the excellent pharmacological activity of bufadienolide, (cardio)toxicity is a non-negligible drawback (Li et al., 2015). In addition, as a specific inhibitor of Na^+^/K^+^ ATPase, bufadienolide would result in serious side effects, including cardiac dysfunction, conduction block, and arrhythmia (Bagrov et al., 2009). It is this serious side effect that severely limits the further pharmacological application of bufadienolide. Therefore, researchers have lit a way to discover bufadienolide derivatives with reserved activity and reduced side effects by performing structure modification *via* biosynthesis.

### Plant suspension culture cell transformation

It is a promising way to employ plant suspension culture cells to perform desired structure modification. A series of works have been reported for bufadienolide structural modification *via* plant suspension culture cells. Ye performed biotransformation of cinobufagin *via* suspension culture cells originated from Catharanthus roseus and Platycodon grandiflorum. Three cinobufagin derivatives, namely 3-*epi*-desacetylcinobufagin, desacetylcinobufagin and 1-hydroxyyl desacetylcinobufagin, were obtained (Ye et al., 2003). Xue also modified telocinobufagin by utilizing Catharanthus roseus suspension culture cells, and a new bufadienolide namely 3-*epi*-telocinobufagin was produced. Three bufadienolides, including bufatalin, gamabufalin and telocinobufagin, were biotranformed by Zhang *via* Saussurea involucrate suspension culture cells. Fortunately, eleven bufadienolide derivatives were obtained, which strongly supported the practicability of plant suspension culture cell transformation for bufadienolide structure modification (Zhang et al., 2011).

### Microbial transformation

Besides the plant suspension culture cell approach, microbial transformation is also a promising alternative. *Alternaria alternata* was employed by Ye et al. to perform the biotransformation of cinobufagin (Ye and Guo 2008), and six cinobufagin derivatives were obtained. After reaction condition optimization, cinobufagin could be totally converted to the major transformation product 12*β*-hydroxy-cinobufagin. Importantly, Ma discovered that *Fusarium solani* could achieve an isomerization reaction for bufadienolide. In detail, it could convert bufadienolide with 3β-hydroxyl configuration to 3α-hydroxyl configuration, and selectively oxidize the 3β-hydroxyl, instead of 3α-hydroxyl, to ketone (Ma et al., 2007).

The nature of the above two approaches is to employ the corresponding enzymes therein with satisfied substrate heterogeneity for bufadienolide structure modification. The operation simplicity and mildness of reaction conditions made them effective approaches to replace chemical synthesis. Nonetheless, there existed obvious drawbacks of these transformation approaches. Of which the most annoying aspect is that these transformations did not exhibit any selectivity to the desired targets due to too many enzymes involving the transformation. This also resulted in the relatively low yield of desired products. For example, only 2% of yield was obtained for cinobufagin biotransformation *via* plant suspension cells. Therefore, both the above two approaches exhibit some limitations and could not be applied to an industrial scale (Yue et al., 2016).

### Enzyme-catalyzed glycosylation

To obtain structurally modified bufadienolides with reduced toxicity, glycosylation may be the first choice with a bright future. In comparison to conventional chemical synthesis, laborious protection/deprotection processes are avoided, and the process is more eco-friendly. Meanwhile, in contrast to plant suspension cell and microbial transformation, enzyme catalytic glycosylation is more efficient and exhibits perfect regional or stereoselectivity, allowing it to be directly exploited to produce structure-modified bufadienolides with the desired property (Zhang et al., 2006). Currently, enzyme catalytic glycosylation has become a focal point of study for bufadienolide structure modification (Elshahawi et al., 2015).

In 2007, Thorson completed the first enzyme catalyzed glycosylation of cardiac steroids. They discovered an actinomycete-sourced glycosyltransferase OleD. After high-throughput screening, a mutant named ASP with high substrate heterogeneity was further discovered (Williams et al., 2007). At the same time, Thorson dug the catalysis property of glycosyltransferase OleD/ASP. It was found that OleD/ASP did not exhibit steroid core site selectivity, and both C-3 and C-12 positions could be glycosylated without any differentiation. Accordingly, Zhu skillfully employed a pair of bufadienolides with different configuration of hydroxyl group at the C-3 position to further explore the stereoselectivity of OleD/ASP. It was revealed that OleD/ASP exhibited highly stereoselectivity: bufadienolide with 3*β*-OH configuration could be glycosylated, while it was powerless to the bufadienolide with 3*α*-OH configuration (Zhu et al., 2018).

**FIGURE 6:**
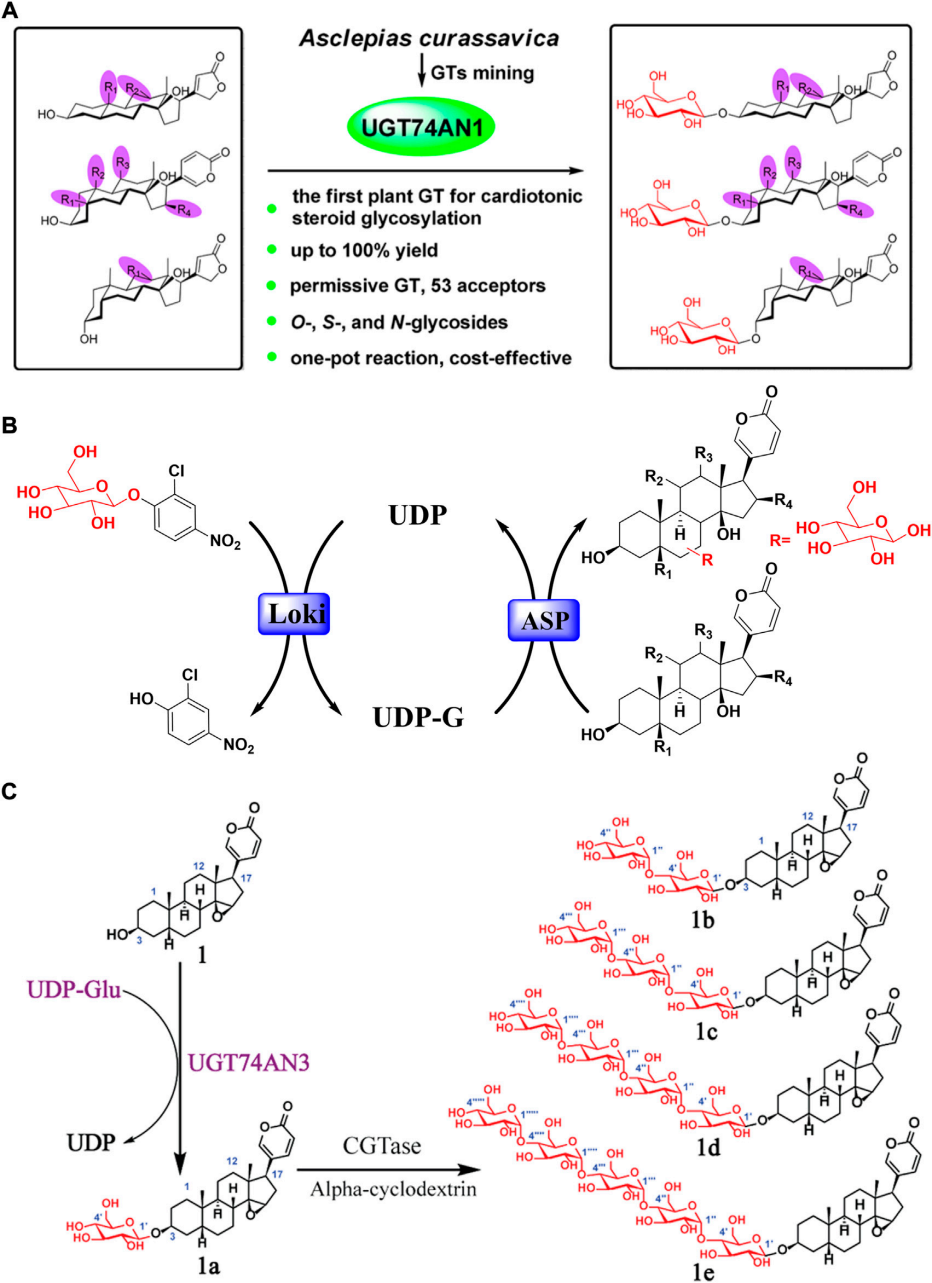
Glycosylation structure modification of bufadienolide: **(A)** UGT74AN1 catalyzed glycosylation of bufadienolide with regiospecificity; **(B)** The typical one-pot glycosylation system for bufadienolide; **(C)** Bufadienolide derivatives linked with sugar chains of varied length at the C-3 position obtained by enzyme catalyzed glycosylation.

Promisingly, Wen excavated a glycosyltransferase named UGT74AN1 from *Asclepias curassavica*, and it could conduct the glycosylation of bufadienolide specifically at C-3 position (Wen et al., 2018) (Figures 6A,B). UGT74AN1 is the first reported steroid glycosyltransferase to generate 3-O-*β*-D-glucoside of bufadienolide with high efficiency and regiospecificity. Furthermore, Huang developed a one-pot enzymatic system coupling UGT74AN3 and CGTase with high efficiency to generate bufadienolides linked with sugar chains of varying lengths at the C-3 position (Huang et al., 2019) (Figure 6C). Importantly, the glycosylation products displayed improved inhibitory efficacy with specificity for the Na^+^/K^+^ ATPase α2 subunit.

In addition, Ye modified bufadienlide at the C-3 position *via* glycosyltransferase Yjic1. It gave a mono-glycoside (bufalin 3-O-*β*-D-glucoside) and a di-glycoside (bufalin 3-O*-β*-D-glccosyl (1→2)-*β*-D-glucoside) with satisfying yields. Compared to the substrate, the solubility of corresponding glycosides was increased by 25-fold and 102-fold, respectively (Li et al., 2017). Brilliantly, Fu first reported the glycosylation of bufadienolide mixtures by glycosyltransferase Yjic1. The subsequent zebrafish embryo toxicity experiment revealed that the toxicity of glycosylated bufadienolides were much less than the corresponding aglycone (Fu et al., 2019).

## Conclusion

The review has highlighted toad-sourced bufadienolides and their anti-inflammatory activity. Based on whether the C-3 hydroxyl group is substituted or not, two types of toad-derived bufadienolide (free or conjugated bufadienolide) were distinguished. And the structural diversity, as well as their MS fragmentation principles, have also been primarily summarized, laying a foundation for future analysis. In addition, the remarkable Na^+^/K^+^ ATPase inhibitory and anti-inflammatory efficacy reported for bufadienolide were also briefly highlighted. During the research process, the serious toxicity pushed the development of structural modification of bufadienolide *via* biotransformation for side effects reduction. It lighted a promising way for broadening the structural diversity and enhancing the pharmacological activity of bufadienolide. The current evidence of bufadienolide we summarized would provide a scientific basis for future in-depth studies and perspectives of potential drug candidate discovery.
